# Spontaneous Hepatic Hemorrhage: An Unexpected Complication From Enoxaparin

**DOI:** 10.7759/cureus.33371

**Published:** 2023-01-04

**Authors:** Abhishrut Jog, Vijil Rajan, Charbel Ishak, Maryam Soliman

**Affiliations:** 1 Pulmonary Medicine, BronxCare Health System, Bronx, USA; 2 Internal Medicine, BronxCare Health System, Bronx, USA; 3 Interventional Radiology, BronxCare Health System, Bronx, USA; 4 Pulmonary and Critical Care Medicine, BronxCare Health System, Bronx, USA

**Keywords:** acute pancreatitis, ct guided drainage, hepatic congestion, anticoagulation, spontaneous hepatic hemorrhage

## Abstract

Spontaneous hepatic hemorrhage (SHH) is a rare condition that occurs due to a breach in the liver parenchyma in the absence of an external cause, most commonly from hepatocellular cancer. If a solid liver lesion is absent, then it has been linked with diffuse hepatic diseases or systemic diseases. Although SHH has been linked with the use of warfarin, it has not been thus far linked with enoxaparin. SHH can present with non-specific symptoms, and lab parameters can reveal substantial drops in hemoglobin. It is diagnosed most commonly with computed tomography (CT) imaging and conservative treatment is effective in the majority of cases. We present one such rare case of SHH.

## Introduction

Spontaneous hepatic hemorrhage (SHH) is a rare condition that occurs due to a breach in the liver parenchyma in the absence of an external cause [1]. The most common cause of SHH (10%) is hepatocellular carcinoma (HCC), followed by other causes such as benign liver lesions (hemangioma, adenoma, biliary cystadenoma), malignancies, amyloidosis, fatty liver of pregnancy, and connective tissue disorders such as lupus erythematosus [2,3]. SHH due to warfarin has been reported, however, isolated use of enoxaparin has not been linked with it [4].

We present a rare case of SHH and describe the progression of the disease, laboratory and radiological features and discuss the various pathophysiological mechanisms behind its occurrence.

## Case presentation

**Figure 1:**
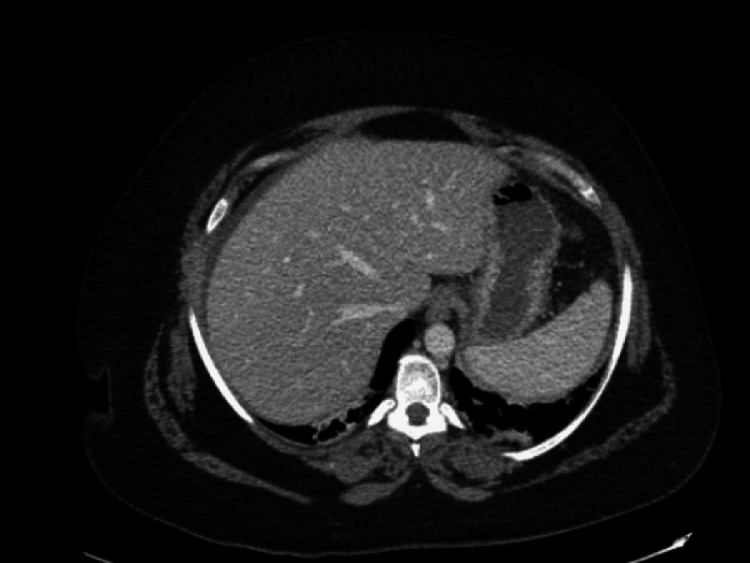
CT scan of the abdomen at admission. Note the normal liver parenchymal features. CT: computed tomography

A 45-year-old woman presented with increasing abdominal pain, distension, nonbilious vomiting, and constipation for three days. The diagnostic evaluation revealed an elevated lipase of 1050 U/L (Table 1) and an edematous pancreas on computed tomography (CT) imaging consistent with acute pancreatitis. Of note, the liver was noted to be normal in this CT scan (Figure 1).

**Table 1:** Blood parameters at admission and at day 19. Note the drop in hemoglobin and the elevation of liver enzymes.

Parameters	Results (Day 1)	Results (Day 19)	Reference
Hemoglobin	14.4 g/dl	6.7 g/dl	12.0-16.0 g/dl
Platelet	183 k/ul	130 k/ul	150-400 k/ul
White blood cell	10.4 k/ul	6.4 k/ul	4.8-10.8 k/ul
Sodium	136 mEq/L	138 mEq/L	135-145 mEq/L
Potassium	4.7 mEq/L	4.2 mEq/L	3.5-5.0 mEq/L
Blood urea nitrogen	15.0 mg/dl	54 mg/dl	6.0-20 mg/dl
Creatinine	0.9 mg/dl	1.5 mg/dl	0.5-1.4 mg/dl
Lipase	1050 U/L	-	< 61 U/L
Aspartate transferase	31 U/L	1595 U/L	9-36 U/L
Alanine transferase	32 U/L	1676 U/L	5-40 U/L
International normalized ratio	0.93	1.40	0.85-1.14
Prothrombin time	11.0 seconds	17.1 seconds	9.9-13.3 seconds
Activated partial thromboplastin time	24 .6 seconds	29.1 seconds	27.2-39.6 seconds

She was started on intravenous fluids (IV) and admitted to the Intensive Care Unit (ICU). Subsequently, the patient developed acute respiratory distress syndrome (ARDS) and was intubated on day 3 of admission. The ventilator was set as per ARDS protocol and a high respiratory rate was used. CT angiogram revealed a right-sided pulmonary embolus for which she was initiated on subcutaneous enoxaparin. She required IV nicardipine for elevated blood pressure during this period. She was noted to have a positive fluid balance of 12 liters in four days and was administered IV furosemide to assist in diuresis. By day 13, the patient’s pulmonary status had improved and she was extubated. A week later, the patient became tachycardic and hypoxic. Labs revealed severely elevated liver enzymes and an acute hemoglobin drop from 9.1 gm/dl to 6.7 gm/dl. Prothrombin time and activated partial thromboplastin time were 17.1 seconds and 29 seconds respectively. The international normalized ratio (INR) was 1.40 and fibrinogen was 393 mg/dl (Table 1).

**Figure 2:**
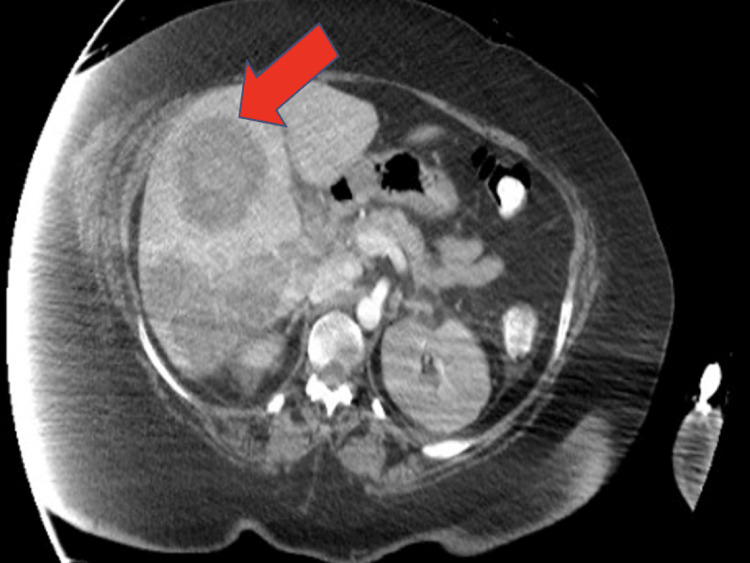
CT abdomen on day 19 showing multiple hyperdense liver lesions. The red arrow points to the largest collection. CT: computed tomography

The patient was re-intubated for hypoxia and transfused two units of blood. CT abdomen revealed the development of multiple hyperdense regions within the liver parenchyma - the largest being 6 cm in diameter (Figure 2).

**Figure 3:**
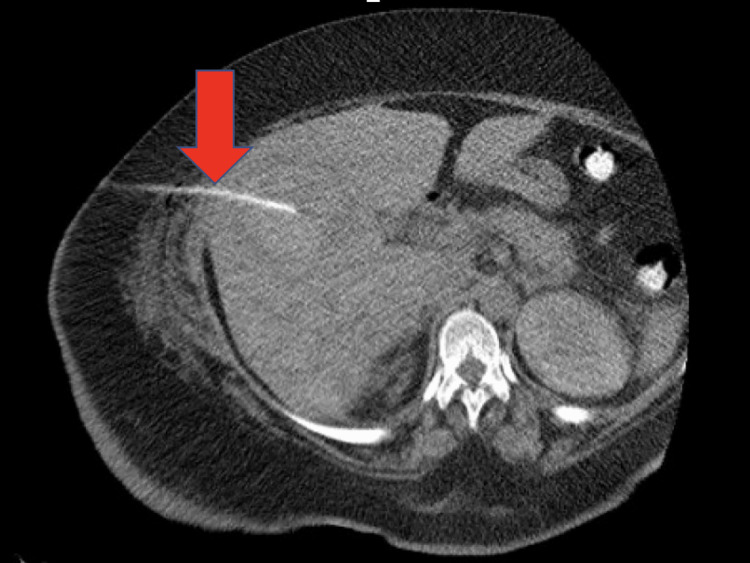
CT-guided drainage of hepatic collection. The red arrow shows the needle. CT: computed tomography

**Figure 4:**
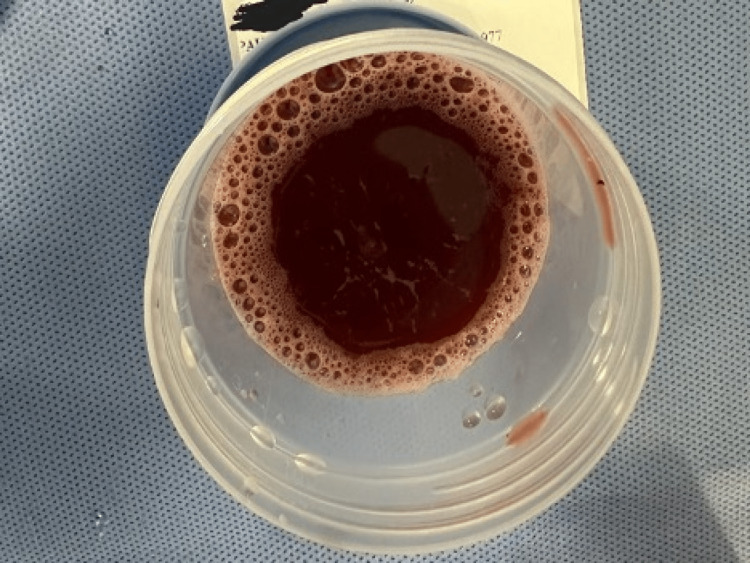
Grossly hemorrhagic fluid obtained with CT guidance. CT: computed tomography

The enoxaparin was held. Interventional radiology (IR)-guided drainage of the lesions revealed grossly hemorrhagic fluid (Figure 3 and Figure 4).

Fluid studies revealed a white blood cell (WBC) count of 20 cells/mm^3^ and a red blood cell (RBC) count of 300000 mil cells/mm^3^ (Table 2).

**Table 2:** Hepatic fluid analysis.

Parameters	Results
Color	Red
White blood cell	20 cells/mm^3^
Red blood cell	300000 mil cells/mm^3^
Segmented cell count	72%
Lymphocyte count	28%
Aerobic culture	negative
Mycobacterial culture	negative
Cytology	Negative for malignancy

Cultures and cytology of the drained fluid were negative. The patient was managed conservatively. No further drop in hemoglobin was noted and she did not require a further blood transfusion. She was subsequently extubated and was eventually discharged home with a hemoglobin of 9.9 gm/dl. She was followed up a month later and found to have returned to her daily activities.

## Discussion

SHH accounts for around 1% of the admissions in specialized liver centers. The high prevalence of HCC results in it being the most common cause of SHH and it is the presenting feature in 10% of HCC cases [3]. Warfarin has been linked with SHH [4]. Enoxaparin has been known to cause hemorrhagic complications in 6.5% of cases within 30 days of initiation [5]. However, its isolated use has not been known to cause liver bleed, in our knowledge.

The pathogenesis of hepatic parenchymal bleeding varies with the etiology, and in most cases is multifactorial [3]. In connective tissue diseases, the SHH is due to the poorly supported or weakened tissue being subjected to physiological events such as blood pressure fluctuations or respiratory movement. In hepatic tumors, the SHH may be from bleeding from a parasitic vessel such as the inferior phrenic artery, bleeding from neovascularization within the tumor, venous congestion, a decrease of von Willebrand factor, and changes in the vascular endothelium. Rapidly growing tumors are known to cause tears in the surrounding hepatic parenchyma [6].

Interestingly, although the incidence of SHH is not increased in end-stage liver failure, coagulopathy, which is a consequence of liver disease, has been shown to initiate and maintain SHH. On autopsy of patients who died with coumadin-derivative toxicity, the finding was of dilatation of sinusoids and structural disorganization to frank parenchymal hemorrhage and subcapsular ecchymoses [4].

In our case, there is speculation that a combination of these mechanisms played a part. The patient was seen to have a fatty liver in the CT scan. The patient underwent a rapid enlargement of this fatty liver during admission. The ultrasound imaging from the day of admission compared with 12 days into admission showed a size increase of 4 cm and reached 20 cm at the time of the hemorrhage. The reason for this is probably hepatic congestion from IV fluid administration for pancreatitis. In pathophysiology similar to rapid tumor growth causing the parenchymal tear, the rapid hepatic growth of an already vulnerable liver led to multiple parenchymal tears in our case. Additionally, the patient was treated with IV nicardipine for high blood pressure readings in the peri-extubation period, which caused fluctuations in blood pressure. The hepatic vasculature being stretched as such was unable to withstand the fluctuating blood pressure. Respiratory variations of the patient on the mechanical ventilator set at a high respiratory rate for ARDS contributed to the worsening of the shearing forces. Lastly, enoxaparin was used for the pulmonary embolus. The additional use of anticoagulation was an incitement for the bleeding.

Although not described commonly within the liver, peripancreatic necrosis and hemorrhage are seen in necrotizing pancreatitis [7]. However, in our case, the hepatic bleed manifested almost three weeks after the diagnosis of acute pancreatitis. Furthermore, the CT scan that diagnosed the bleeding did not show any necrotic features in the pancreas, making this explanation less likely, albeit possible.

The presenting features of SHH are nonspecific in 90% of cases, with abdominal pain, malaise, and vomiting being the most common and hematemesis, fever, jaundice, and shock being less common [8]. Hence a high index of suspicion is needed for diagnosis. In our case, the patient presented with abdominal pain and vomiting. In addition, she was noted to have a precipitous drop in hemoglobin.

CT scan of the abdomen with contrast extravasation is the gold standard diagnostic test for SHH. However, in half of the cases, the imaging can show just intrahepatic hematoma, as in our case [8]. Further confirmation was sought with IR-guided drainage of the lesion, where frank blood was seen, with an RBC count of 300,000 mil cells/mm^3^, confirming the diagnosis as SHH. Twenty percent of cases of SHH are diagnosed intra-operatively due to the instability of the cases at presentation [9].

SHH is managed initially with hemostasis and then subsequently with definitive treatment. The following approaches for hemostasis are used: conservative approach (resuscitation and blood product replacement), trans-arterial embolization, hepatic artery ligation, segmental hepatic artery ligation, packing/plication, and resection [3]. Conservative management results in the achievement of hemostasis in over half of the cases [10], and this was the course taken in our case. In our case, the SHH stabilized and did not continue to expand. She was resuscitated with fluids and blood product replacement. Definitive treatment is based on the underlying cause.

## Conclusions

SHH is a rare occurrence in the absence of a solid hepatic lesion or severe liver disease. Anticoagulation, rapid enlargement, rapid respiration, changes in blood pressure, and possibly peri-pancreatic inflammation from pancreatitis are all possible contributing factors.

CT imaging is a valuable resource in diagnosing intra-abdominal bleeding including hepatic bleeds. It should be sought in most cases that present with a precipitous drop in hemoglobin without an obvious external bleeding source. CT-guided drainage and lab testing of the fluid prove the diagnosis. Conservative management is effective.
